# A Shear-Mode Magnetoelectric Heterostructure with Enhanced Magnetoelectric Response for Stray Power-Frequency Magnetic Field Energy Harvesting

**DOI:** 10.3390/mi13111882

**Published:** 2022-11-01

**Authors:** Wei He

**Affiliations:** School of Information Engineering, Baise University, Baise 533000, China; weiheky@yeah.net

**Keywords:** magnetoelectric heterostructure, enhanced magnetoelectric response, power-frequency magnetic field, shear piezoelectric effect, theoretical model

## Abstract

This paper devises a magnetoelectric (ME) heterostructure to harvest ambient stray power-frequency (50 Hz or 60 Hz) magnetic field energy. The device explores the shear piezoelectric effect of the PZT-5A plates and the magnetostrictive activity of the Terfenol-D plates. The utilization of the high-permeability films helps to enhance the magnetoelectric response to the applied alternating magnetic field. A theoretical model is developed based on the piezomagnetic and piezoelectric constitutive equations as well as the boundary conditions. The ME response of the device is characterized theoretically and experimentally. The measured ME voltage coefficient attains 165.2 mV/Oe at the frequency of 50 Hz, which shows a good agreement with the theoretical result. The feasibility for extracting energy from the 50 Hz magnetic field is validated. Under an external alternating magnetic field of 30 Oe, a maximum power of 8.69 μW is generated across an optimal load resistance of 693 kΩ. Improvements of the ME heterostructure are practicable, which allows an enhancement of the ME voltage coefficient and the maximum power by optimizing the structural parameters and utilizing PMN-PT with a higher shear-mode piezoelectric voltage coefficient (g_15_).

## 1. Introduction

With the development of the wireless sensor networks (WSNs), higher demands on the power supply of the sensor nodes have been put forward. The traditional batteries cannot satisfy the requirement on the long-term operation of the sensor nodes due to the limited energy. A prospective method to address this issue is using energy harvesting technology, which extracts energy from ambient energy sources. Researchers have conducted investigations on different energy sources, such as sunlight [1], sound [2,3], heat [4,5], human motion [6,7,8], and mechanical vibration [9,10,11], and a variety of schemes for different energy sources have been developed [12,13,14].

The stray power-frequency (50 Hz or 60 Hz) magnetic field originating from electric power systems (e.g., power lines, busbars) is another promising energy source for energy harvesting. The feasibility for harvesting energy from a 50 Hz or 60 Hz magnetic field has been explored. A typical scheme is utilizing a current transformer [15], but the intrinsic shortcomings (e.g., saturation, sharp outputs) render the device unpractical. Mechanisms based on piezoelectric cantilever beams have been presented to extract energy from the power-frequency magnetic field [16,17]. To achieve maximum outputs, the devices need to be tuned to the targeted frequency of 50 Hz or 60 Hz.

Magnetoelectric (ME) laminate composites have been proved to be effective in magnetoelectric transduction [18,19]. Cantilever-based resonant structures using ME laminate composites have been devised to harvest 50 Hz magnetic field energy from power lines [20,21], and the resonant frequency also needs to be adjusted due to the nonlinear magnetic force. In real applications, a non-resonant energy harvesting device might be preferred for a 50 Hz or 60 Hz magnetic field. The conventional ME laminate composites (e.g., MP and MPM structures) can be used as non-resonant devices to extract such low-frequency (50 Hz or 60 Hz) magnetic field energy, but it is estimated that the harvested energy is quite low. ME structures have been devised to enhance the ME responses [22,23,24,25], including improving the piezomagnetic coefficient [22], exploring the shear piezoelectric effect [23,24], and using a mechanically mediated device [25], but the ME responses at the power frequency (50 Hz or 60 Hz) are not investigated in the above literature.

In this paper, a ME heterostructure with an enhanced ME response is presented. Shear piezoelectric effect is induced due to the shrinkage/elongation activity of the Terfenol-D plates and the maintaining of retaining plates. Theoretical analysis has been performed to predict the ME voltage coefficient and the maximum power, and the theoretical results match the experimental results well. The feasibility for harvesting the 50 Hz magnetic field is verified, and the output power can be further improved by structure and material optimization.

## 2. Magnetoelectric Heterostructure

**Figure 1:**
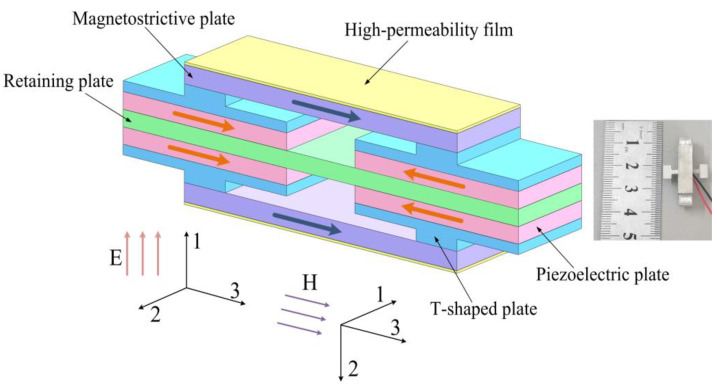
Schematic diagram and picture of the proposed shear-mode ME heterostructure.

**Figure 2:**
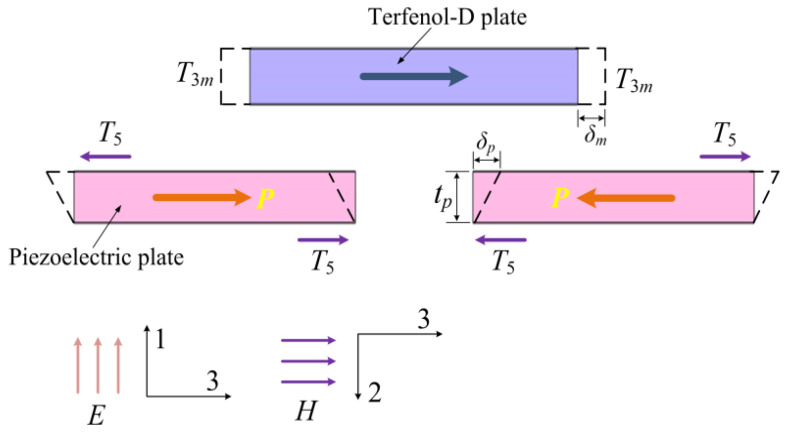
Deformations of the Terfenol-D plates and the piezoelectric plates under operation.

Figure 1 shows the schematic diagram of the proposed shear-mode ME heterostructure. Two coordinate systems are used for the piezoelectric plates and the magnetostrictive plates, which respectively apply to the piezoelectric and piezomagnetic constitutive equations. The device consists of four shear-mode piezoelectric plates (PZT-5A), four T-shaped retaining plates, two magnetostrictive plates (Terfenol-D), a rectangular retaining plate, and two high-permeability films bonded on the surfaces of the Terfenol-D plates. A longitudinal shift of the Terfenol-D plate is induced when an alternating magnetic field (along the 3-direction) is applied due to the magnetostrictive effect. The stress of the Terfenol-D plate is transmitted to the PZT-5A plate by the T-shaped retaining plate. Shear stress is then produced on the PZT-5A plate as a result of the effect of the T-shaped and rectangular retaining plates. The PZT-5A plate produces a voltage output owing to the shear piezoelectric effect, as shown in Figure 2. The employing of the high-permeability films helps to enhance the piezomagnetic coefficient as well as the ME voltage coefficient, which can potentially improve the output power for an ambient low-frequency magnetic field (e.g., stray 50 Hz power-frequency magnetic field) energy harvesting.

After bonding a high-permeability film on the surface of the Terfenol-D plate, the internal effective magnetic field of the magnetostrictive plate can be expressed as

(1) $$H_{eff}\approx \frac{H_{dc}+H_{f}}{1+\chi N_{d}},$$

where *H_dc_* is the applied bias magnetic field, *H_f_* is the additional magnetic field produced by the film, which can be calculated using equivalent magnetic charge method [26], *χ* is the magnetization coefficient, and *N_d_* is the demagnetizing factor. The effective piezomagnetic coefficient is given by [27,28]

(2) $$d_{33,m}^{eff}=\frac{2\lambda_{s}{\left(\mu_{r}-1\right)}^{2}H_{eff}}{M_{s}^{2}}\left\{ \begin{array}{ll} 1-\tanh(\frac{T}{T_{s}}) & \left(T\geq 0\right)\\ 1-\frac{\tanh(2T/T_{s})\;}{2} & \left(T<0\right) \end{array}\right.,$$

(3) $$T=\frac{-E_{f}E_{m}t_{f}\Delta \varepsilon_{1}}{\left(1-\nu )(E_{f}t_{f}+E_{m}t_{m}\right)},$$

where *λ_s_* is the saturation magnetostrictive coefficient, *μ_r_* denotes the relative permeability, * M_s_* is the saturation magnetization intensity, *T* is the stress exerted on the Terfenol-D plate produced by the high-permeability film, *T_s_* is the saturation stress, *E_f_* and *t_f_* are, respectively, the elastic modulus and thickness of the high-permeability film, *E_m_* and *t_m_* are the elastic modulus and thickness of the Terfenol-D plate, respectively, Δ*ε*_1_ represents the strain of the high-permeability film, and *ν* is the Poisson’s ratio. The piezomagnetic constitutive equations for the Terfenol-D plate are given by [29]

(4) $$S_{3m}=s_{33}^{H}T_{3m}+d_{33,m}^{eff}H_{3},$$

(5) $$B_{3}=d_{33,m}^{eff}T_{3m}+\mu_{33}^{T}H_{3},$$

where *S*_3*m*_ and *T*_3*m*_, respectively, represent the strain and the stress, $s_{33}^{H}$ is the elastic compliance coefficient, *H*_3_ and *B*_3_ denote the magnetic field and magnetic induction density, respectively, and $\mu_{33}^{T}$ is the permeability. Considering one shear-mode PZT-5A plate shown in Figure 2, only the electric displacement *D*_1_ and the electric field *E*_1_ are taken into account based on the polarization and the configuration of the electrodes, and the constitutive equations are given by [30]

(6) $$S_{5}=s_{55}^{D}T_{5}+g_{15}D_{1},$$

(7) $$E_{1}=-g_{15}T_{5}+\frac{D_{1}}{\varepsilon_{11}^{T}},$$

where *S*_5_ and *T*_5_, respectively, represent the shear strain and stress, $s_{55}^{D}$ denotes the elastic compliance coefficient (under constant electric displacement), *g*_15_ represents the piezoelectric voltage coefficient, and $\varepsilon_{11}^{T}$ is the permittivity. Based on the model shown in Figure 2, one obtains the following boundary condition by considering the balance of the forces on a T-shaped retaining plate

(8) $$A_{m}T_{3m}=A_{p}T_{5},$$

where *A_m_* denotes the cross-sectional area of the Terfenol-D plate, *A_p_* is the area of one electrode surface of the PZT-5A plate. In Figure 2, it is assumed that the retaining plates are rigid and perfectly bonded with the other components of the device. The strains of the Terfenol-D plate and the PZT-5A plate are obtained, which are respectively given by

(9) $$S_{3m}=\frac{2\delta_{m}}{l_{m}},$$

(10) $$S_{5}=-\frac{\delta_{p}}{t_{p}},$$

where *l_m_* is the length of the Terfenol-D plate (except the bonding parts with the T-shaped retaining plates), and *t_p_* is the thickness of the PZT-5A plate. The following boundary can be applied by considering *δ_m_* = *δ_p_*, which is given by

(11) $$S_{3m}l_{m}+2S_{5}t_{p}=0.$$

To obtain the ME voltage coefficient, it is assumed that *D*_1_ = 0 (open-circuit boundary) in Equations (6) and (7), the ME voltage coefficient is obtained by combining Equations (4), (6)–(8), and (11), which is given by

(12) $$\alpha_{V}=\left|\frac{V_{1}}{H_{3}}\right|=\left|\frac{E_{1}t_{p}}{H_{3}}\right|=\frac{d_{33,m}^{eff}g_{15}A_{m}l_{m}t_{p}}{s_{33}^{H}A_{p}l_{m}+2t_{p}s_{55}^{D}A_{m}}.$$

The four PZT-5A plates are connected in parallel to attain a large total equivalent capacitance *C_total_* for energy harvesting from the power-frequency magnetic field. Assuming that the power power-frequency magnetic field *H*(*t*) = *H*_0_cos(*ωt* + *φ*), the open-circuit voltage is given by

(13) $$V_{open}(t)=a_{V}H(t)=a_{V}H_{0}\cos(\omega t+\varphi ),$$

where *H*_0_ denotes the amplitude of the alternating magnetic field, *ω* represents the angular frequency, *ω =* 2*πf*, *f* = 50 Hz or 60 Hz, and *φ* is the phase angle. The root mean square (RMS) value of the open-circuit voltage can be expressed as

(14) $$V_{RMS}=\frac{\sqrt{2}\alpha_{V}H_{0}}{2},$$

When a load resistance *R* is connected to the output of the ME heterostructure, the voltage and power are expressed as

(15) $$V_{Load}=\left|\frac{V_{RMS}R}{(1/j\omega C_{total})+R}\right|=\frac{V_{RMS}R\omega C_{total}}{\sqrt{1+(R\omega C_{total}{)}^{2}}},$$

(16) $$P_{Load}=\frac{V_{Load}^{2}}{R}=\frac{V_{RMS}^{2}R\omega^{2}C_{total}^{2}}{1+(R\omega C_{total}{)}^{2}},$$

where *C_total_* denotes the total capacitance, which is given by

(17) $$C_{total}=\frac{4A_{p}\varepsilon_{0}\varepsilon_{r}}{t_{p}}.$$

where *ε_r_* is the relative dielectric constant. The maximal output power is obtained at *R_opt_* = 1/(*ωC_total_*), which is given by

(18) $$P_{\max}=\frac{\omega C_{total}V_{RMS}^{2}}{2}=\frac{{\left(d_{33,m}^{eff}\right)}^{2}g_{15}^{2}A_{m}^{2}l_{m}^{2}t_{p}^{2}\omega C_{total}H_{0}^{2}}{4{\left[s_{33}^{H}A_{p}l_{m}+2t_{p}s_{55}^{D}A_{m}\right]}^{2}},$$

The maximal output power *P_max_* is directly proportional to the square of $d_{33,m}^{eff}$ and the square of $g_{15}$, which is of great significance for the design of the shear-mode ME heterostructure.

## 3. Results and Discussions

**Figure 3:**
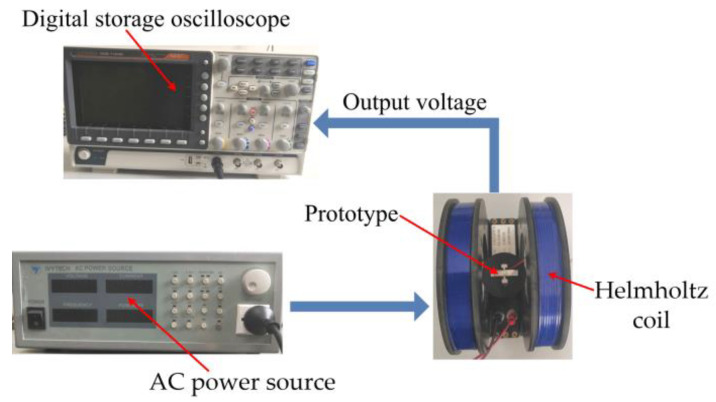
Measurement principle for the induced voltages of the fabricated prototype.

To validate the performances of the presented ME heterostructure, experiments were performed using a fabricated prototype. The prototype was fabricated according to the following procedure. (1) The components of the heterostructure were cleaned utilizing propanone. (2) The rectangular retaining plate (29 mm × 6 mm × 1.2 mm) and the piezoelectric plates (shear-mode PZT-5A with the dimension of 12 mm × 6 mm × 1 mm for each plate) were bonded using insulate epoxy adhesive. (3) The T-shaped retaining plates (aluminium alloy 6061) were bonded with the PZT-5A plates. (4) The magnetostrictive plates (Terfenol-D with the dimension of 20 mm × 6 mm × 2 mm for each plate and *l_m_* = 14 mm) are bonded with the T-shaped retaining plates using insulate epoxy adhesive according to Figure 1. (5) The high-permeability films were glued together with the Terfenol-D plates. For each T-shaped retaining plate, the dimension of the base is 12 mm × 6 mm × 0.8 mm and the size of the centrally bulged part is 3 mm (along the 3-direction) × 6 mm (along the 2-direction in the coordinate system of the PZT-5A) × 0.8 mm. The other parameters for the calculation of the ME voltage coefficient are $d_{33,m}^{eff}$ = 1.415 × 10^−8^ m/A (*t_f_* = 30 μm), *g*_15_ = 38.2 × 10^−3^ Vm/N, $s_{33}^{H}$ = 4 × 10^−11^ m^2^/N, $s_{55}^{D}$ = 25.2 × 10^−12^ m^2^/N. The ME voltage coefficient is then estimated to be ~176.6 mV/Oe using Equation (12). The measuring principle for the output voltages of the prototype is shown in Figure 3. An alternating current (AC) power source is used to drive a Helmholtz coil, which produces an alternating magnetic field for the prototype. The induced voltages were measured utilizing a digital storage oscilloscope.

**Figure 4:**
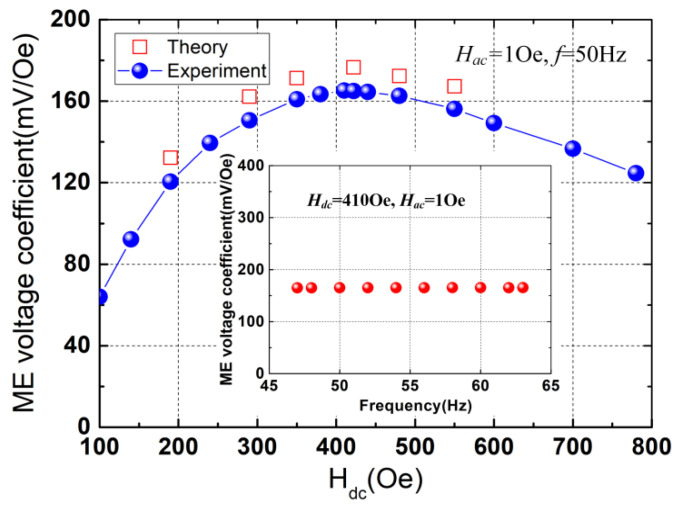
ME voltage coefficient as a function of the bias magnetic field under the alternating magnetic field of 1 Oe peak and at the power frequency of 50 Hz. The inset shows the frequency response of the measured ME voltage coefficient near the power frequencies under an alternating magnetic field of 1 Oe peak.

The ME voltage coefficient was measured for different bias magnetic fields (*H_dc_*) at the power-frequency of 50 Hz and an alternating magnetic field (*H_ac_*) of 1Oe, and the results are plotted in Figure 4. As shown in Figure 4, the ME voltage coefficient exhibits a nearly linear increase for *H_dc_* < 200 Oe and attains a maximum value of 165.2 mV/Oe at the optimal *H_dc_* of 410 Oe, which corresponds to the maximum magnetostriction of the Terfenol-D. The measured ME voltage coefficient is in good agreement with the calculated result (~176.6 mV/Oe). The ME voltage coefficient (165.2 mV/Oe) is larger than those of the traditional ME laminate composites using PZT [31,32,33]. For example, the ME voltage coefficient is 56 mV/Oe for the longitudinal-transverse (LT) mode [31] and is 86 mV/Oe for longitudinal magnetized/longitudinal polarized (L-L) mode [32]. The ME voltage coefficient near the power frequencies (50 Hz and 60 Hz) is shown in the inset of Figure 4, and a flat response is observed. The nearly linear response of the ME voltage coefficient to the bias magnetic field within the low-field range demonstrates the potential application prospect of the proposed device for use in DC magnetoelectric sensors.

**Figure 5:**
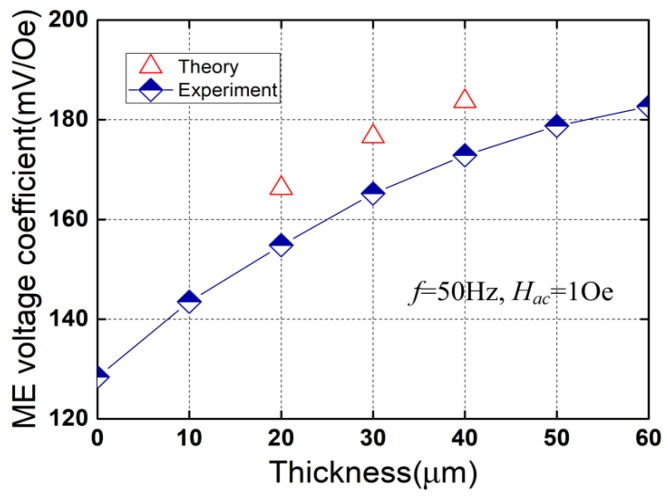
ME voltage coefficient versus the thickness of the high-permeability film.

Figure 5 plots the ME voltage coefficient as a function of the thickness of the high-permeability film (*t_f_*) at the frequency of 50 Hz and under the alternating magnetic field of 1 Oe peak. As shown in Figure 5, the ME voltage coefficient is 128.39 mV/Oe for *t_f_* = 0 (without high-permeability films), and increases from 143.5 mV/Oe to 182.64 mV/Oe as the thickness *t_f_* is increased from 10 μm to 60 μm (Δ*t_f_* = 10 μm). The ME voltage coefficient for *t_f_* = 60 μm is ~1.42 times larger than that for *t_f_* = 0 μm. The theoretical result for *t_f_* = 30 μm is ~176.6 mV/Oe, which is in good agreement with the measured result (165.2 mV/Oe) with the error of ~6.9%. The main reason for the increase in the ME voltage coefficient is presented as follows. With the increasing of the thickness of the high-permeability film (*t_f_*), the amplitude of the stress *T* increases, resulting in an increase in the piezomagnetic coefficient ($d_{33,m}^{eff}$) as well as the ME voltage coefficient (*α_V_*).

**Figure 6:**
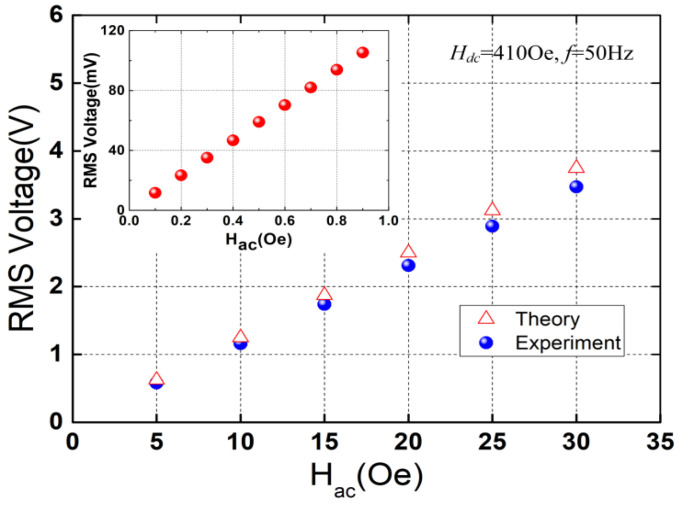
Open-circuit RMS voltage as a function of the applied alternating magnetic field. The inset shows the measured results below 1 Oe.

Figure 6 shows the open-circuit RMS voltage of the ME heterostructure versus the alternating magnetic field (peak value) at the bias magnetic field *H_dc_* = 410 Oe and the power-frequency of 50 Hz. As can be observed from Figure 6, the RMS voltage nearly linearly increases from 0.58 V to 3.47 V when *H_ac_* increases from 5 Oe to 30 Oe (the theoretical RMS voltage increases from ~0.624 V to ~3.75 V). The theoretical values match the experimental results well with the maximum error < 9%. It can be observed from the inset of Figure 6 that the RMS voltage exhibits a good linear relationship with the alternating magnetic field below 1 Oe. The results demonstrate that the proposed ME heterostructure with strong response to the applied alternating magnetic field is feasible to be used as an energy harvester for the stray power-frequency magnetic field.

**Figure 7:**
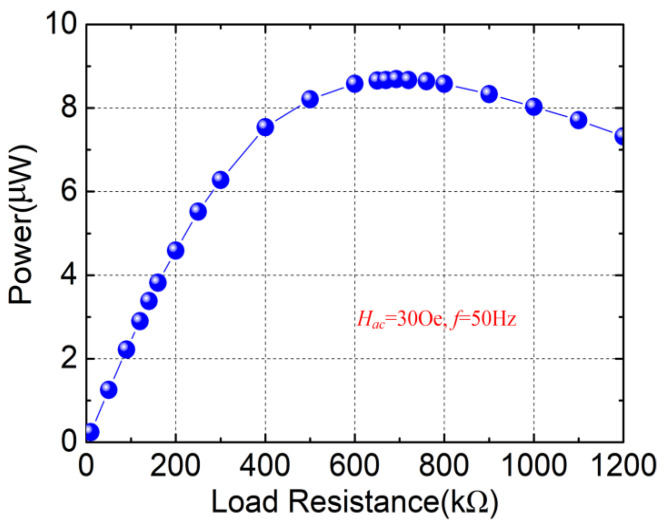
Output power versus load resistance under the alternating magnetic field of 30 Oe.

The load voltages were measured by utilizing different external load resistances under *H_ac_* = 30 Oe (*H_dc_* = 410 Oe), and the powers are obtained based on the following formula

(19) $$P=\frac{V_{L}^{2}}{R_{L}},$$

where *V_L_* is the voltage across the load resistance *R_L_*. The resulting powers for various load resistances are plotted in Figure 7. As can be observed from Figure 7, an approximate linear increase is observed below 200 kΩ. The output power attains a maximum value of 8.69 μW across the load resistance of 693 kΩ. As expected, the power decreases with the load resistance when *R_L_* > 693 kΩ. The theoretical maximum output power obtained from Equation (18) is ~10.05 μW with a matching load resistance of 699 kΩ, which verifies the experimental result.

**Figure 8:**
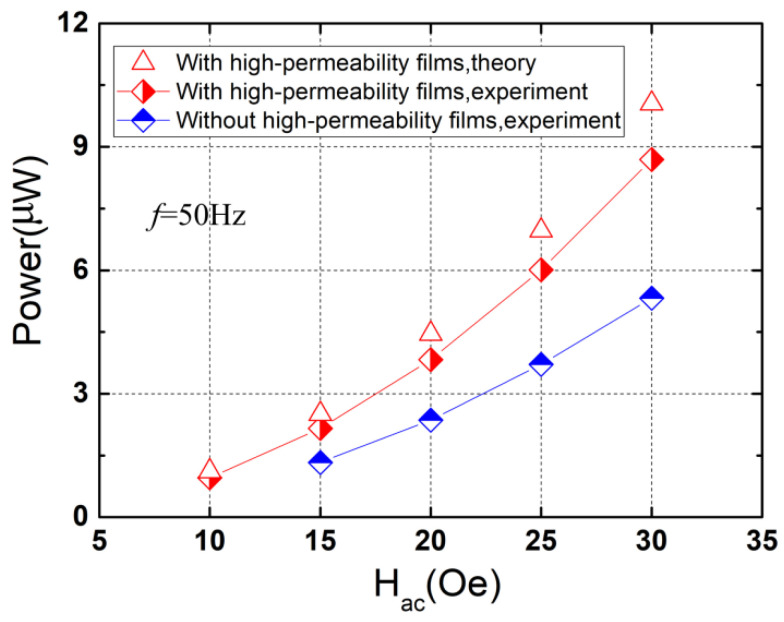
Maximum output power versus the applied alternating magnetic field.

Figure 8 plots the maximum output powers for various alternating magnetic fields. As can be observed from Figure 8, the power of the device with high-permeability films (*t_f_* = 30 μm) increases from 0.96 μW to 8.69 μW nearly in a quadratic relationship when *H_ac_* varies from 10 Oe to 30 Oe (the theoretical power increases from ~1.114 μW to ~10.05 μW). Larger maximum powers can be obtained when the high-permeability films are used (e.g., the maximum power for *t_f_* = 30 μm is ~1.62 times larger than that for *t_f_* = 0 μm under *H_ac_* = 25 Oe), indicating that more 50 Hz magnetic field energy can be converted into electrical energy by using high-permeability films, which is of importance for the design of the non-resonant magnetic field energy harvester.

## 4. Conclusions

In summary, a ME heterostructure exploring the shear piezoelectric effect of the piezoelectric material is proposed to extract energy from the stray power-frequency magnetic field. Four piezoelectric plates are used to improve the total equivalent capacitance. The piezoelectric plates of the device deform in the *d*_15_ mode owing to the magnetostriction of the Terfenol-D, which produce a voltage proportional to the applied alternating magnetic field. The utilization of the high-permeability films helps to improve the piezomagnetic coefficient, which results in an enhancement of the ME voltage coefficient. The working principle under the external alternating magnetic field was stated and a theoretical analysis was conducted. A prototype was fabricated and experimentally characterized. An enhanced ME voltage coefficient of 165.2 mV/Oe is obtained at 50 Hz, which matches the theoretical result well. The feasibility of the device for harvesting 50 Hz magnetic field energy was verified. A maximum power of 8.69 μW is obtained at 30 Oe. Optimization of the ME heterostructure is achievable. The ME voltage coefficient and the maximum power can be further enhanced by optimizing the materials (e.g., adopting shear-mode PMN-PT) and the structural parameters of the device.

## Data Availability

All relevant data are available from the author upon reasonable request.
